# Cystic partially differentiated nephroblastoma in a 74‐year‐old patient

**DOI:** 10.1002/iju5.12357

**Published:** 2021-08-19

**Authors:** Michikata Hayashida, Shoichi Nagamoto, Akihiro Yano, Takayoshi Fu, Naoto Tanaka, Kiichi Hagiwara, Suguru Oka, Kazushige Sakaguchi, Keiichi Kinowaki, Shinji Urakami

**Affiliations:** ^1^ Department of Urology Toranomon Hospital Tokyo Japan; ^2^ Department of Pathology Toranomon Hospital Tokyo Japan

**Keywords:** cystic partially differentiated nephroblastoma, surgical resection, Wilms tumor

## Abstract

**Introduction:**

Cystic partially differentiated nephroblastoma is a multilocular cystic variant of Wilms tumor that always presents in children. However, we encountered an elderly patient with cystic partially differentiated nephroblastoma. Therefore, we report it.

**Case presentation:**

A 74‐year‐old male presented with a left renal tumor detected with ultrasonography. Contrast‐enhanced computed tomography and magnetic resonance imaging revealed a 4 cm multilocular cystic tumor with septa, which suggested multilocular cystic renal cell carcinoma. Therefore, we performed a radical nephrectomy. The definitive diagnosis of cystic partially differentiated nephroblastoma was made with histopathological findings. After the surgical resection, no recurrence has occurred in the past 13 years.

**Conclusion:**

Cystic partially differentiated nephroblastoma can develop in adults, regardless of age. Furthermore, surgical resection can be used as an established treatment option in adult cystic partially differentiated nephroblastoma cases.

**Abbreviations & Acronyms**
CN: cystic nephroma
CPDN: cystic partially differentiated nephroblastoma
CT: contrast‐enhanced computed tomography
MCRCC: multilocular cystic renal cell carcinoma
MRI: magnetic resonance imaging
WT1: Wilms tumor 1

**Keynote message**

Cystic partially differentiated nephroblastoma could develop in adults, regardless of age. Histopathological examination is needed for a definitive diagnosis since preoperative discrimination is difficult. Surgical resection can be used as an established treatment option in adult cases, as well as in children.

## Introduction

CPDN is a rare kidney tumor classified as a multilocular cystic variant of Wilms tumor. Though CPDN primarily presents in children,^1^, ^2^, ^3^ we encountered an elderly patient with CPDN. This is the fifth published adult CPDN case, and our case involves the oldest patient reported so far.^2^, ^3^, ^4^, ^5^ The clinical course of this case can be helpful to guide treatment decisions for CPDN in adults, given the limited number of cases. Herein, we report this case with relevant cases reported in the literature.

## Case presentation

**Fig. 1:**
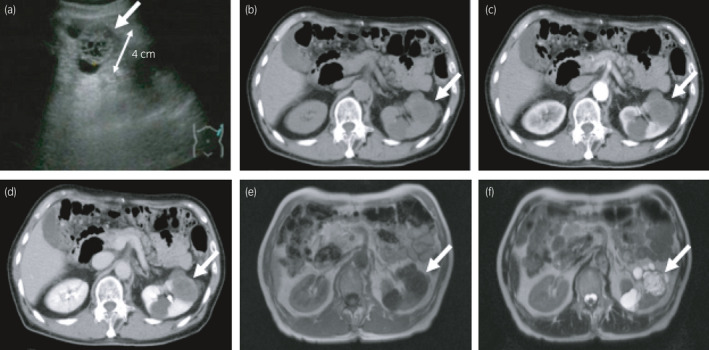
Images of the multilocular cystic tumor with septa (white arrows) at the central pole of the left kidney. (a) An image of ultrasonography, (b) plain CT, (c) contrast‐enhanced CT in an early phase, and (d) contrast‐enhanced CT in a late phase. Septa located in the tumor showed a contrast effect. Images of MRI are displayed in panels e and f. (e) T1‐weighted image (f) T2‐weighed image. The multilocular cystic tumor showed low intensity on the T1‐weighted image and high intensity on the T2‐weighted image.

**Fig. 2:**
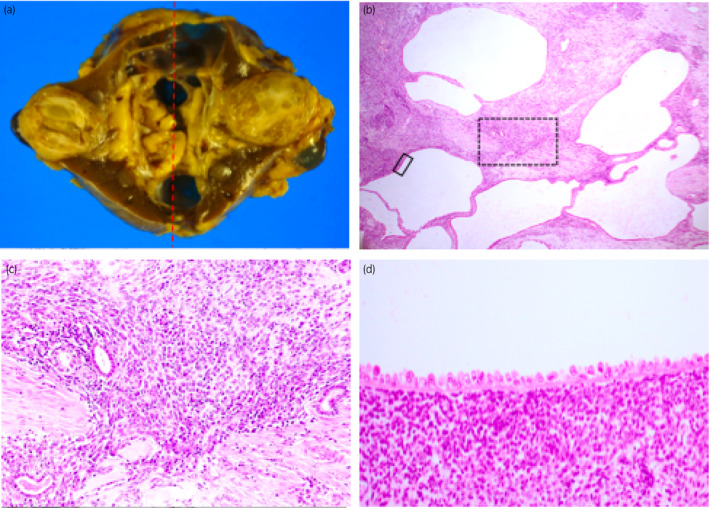
Images of histopathological examination. (a) An image of the resected specimen. A 4 cm, well‐circumscribed, multilocular cystic tumor with septa that had no solid components. Microscopic images of hematoxylin and eosin staining of the specimen are presented in panels b–d. (b) An image in 40× field of view. (c) An image of a septa at 200× field of view (dotted square) at 40× field of view. Immature cells with round or oval nucleus and scanty cytoplasm at the septa, without clear cells, were observed. (d) An image at 400× field of view of the line square in 40× field of view. Hobnail epithelium and mesenchymal cells are visible along with a wall of cysts.

**Fig. 3:**
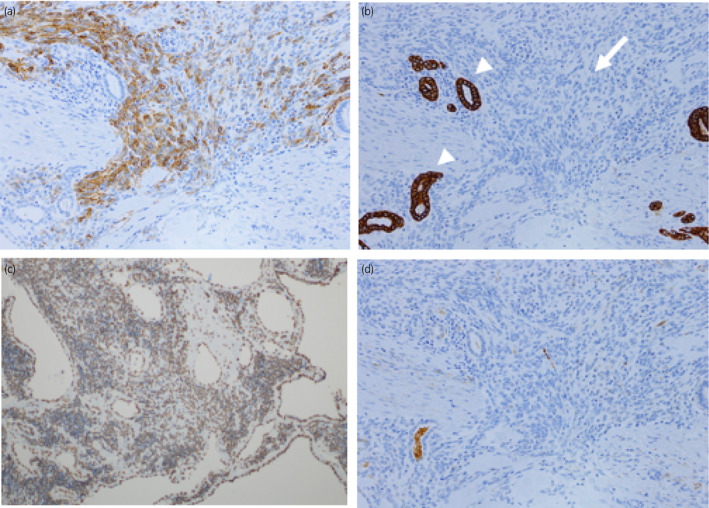
Images of immunopathological examination. CD56, (b) AE1/AE3, (c) PAX2, and (d) Wilms’ tumor 1 (WT1) protein. The specimen was positive for CD56 and PAX2. The epithelial cells were positive (white arrow head) and the mesenchymal cells were negative for AE1/AE3 (white arrow). The specimen was negative for WT1.

A 74‐year‐old male presented with asymptomatic left renal tumor. He underwent ultrasonography every year for postoperative surveillance of gastric cancer. Ultrasonography revealed an appearance of 4 cm multilocular cystic tumor with septa in the middle portion of left kidney (Fig. 1a). Blood and urine tests showed no abnormalities. CT showed contrast‐enhanced septa in the tumor (Fig. 1b–d). MRI revealed a multilocular cystic tumor that showed low and high intensity on T1‐ and T2‐weighted imaging, respectively (Fig. 1e–f). Though the Bosniak grade of the tumor was IIF, we suspected a malignant tumor since the tumor newly appeared within a year. In addition, the patient wanted to remove the tumor. Therefore, a left radical nephrectomy was performed. The resected specimen showed a 4 cm, well‐circumscribed, multilocular cystic tumor with septa that had no solid component (Fig. 2a). Histopathological examination with hematoxylin‐eosin staining found hobnail epithelium and mesenchymal cells along with a wall of cysts; additionally, immature cells with a round or oval nucleus and scanty cytoplasm at the septa, without clear cells, were observed (Fig. 2b–d). Immunopathological examination showed the specimen was positive for CD56 and PAX2, the epithelial cells were positive and the mesenchymal cells were negative for AE1/AE3, and the specimen was negative for the WT1 protein (Figure 3a–d). These results were consistent with those of previous studies on CPDN, thereby confirming the diagnosis of CPDN. The resection margin was negative. After surgical resection, no recurrence has occurred in the past 13 years.

## Discussion

CPDN is a multicystic renal tumor classified as a rare variant of Wilms tumor with unique clinical characteristics.^1^, ^2^ CPDN typically presents in children before 2 years old with male predominance.^6^ Adult CPDN cases are extremely rare with only four cases reported to date.^2^, ^3^, ^4^, ^5^ Details of published cases are summarized in Table 1. Our patient was the oldest among the published cases, suggesting CPDN can develop at any age.

**Table 1:** Details of adult CPDN cases reported in the literature

Author	Year	Sex	Age	Tumor size	Operation performed	Blastema cell	WT1 staining	Outcome
Nagao, et␣al.^4^	1999	M	45	3 cm	Nephrectomy	+	=	No recurrence for 11 months after operation, without adjuvant therapy
Valero Puerta, et␣al.^5^, ^†^	1998	F	21	7 cm	Partial nephrectomy	N/A^‡^	N/A	N/A
Kumar, et␣al.^2^	2001	F	26	7 cm	Nephrectomy	+	N/A	Being followed regularly
Tajima, et␣al.^3^	2015	M	45	3 cm	Partial nephrectomy	−	+	No recurrence for 30 months after operation, without adjuvant therapy
Present case	2021	M	74	4 cm	Nephrectomy	+	=	No recurrence for 13 years after operation, without adjuvant therapy

Information about each patient, tumor, operation performed, pathological findings, and the clinical course is presented from this case and from four cases published in the literature. M, male; F, female.

^†^ This manuscript was written in Spanish. Only the abstract was written in English. Therefore, less information was available about this case compared to the other cases.

^‡^ N/A: not applicable, which means that information could not be obtained from the literature.

The differential diagnoses of renal multicystic tumors in adults are MCRCC, CN, and mixed epithelial and stromal tumors of the kidney.^3^, ^4^ Wilms tumor could also be considered as a differential diagnosis of CPDN; however, Wilms tumor rarely occurs in adults.^3^ Discriminating CPDN from other multicystic tumors on imaging modalities is challenging because their imaging features are similar.^3^ Therefore, histopathological examination is essential to confirm a diagnosis of CPDN. Joshi et␣al. established the histopathological criteria for the diagnosis of CPDN as follows: (1) the lesion is composed entirely of cysts and their septa; (2) it forms a discrete mass that is well‐demarcated from the non‐cystic renal parenchyma; (3) the septa are the only solid portions of the tumor that conform to the outlines of the cysts without solid expansile nodules; (4) the cysts are lined by flattened cuboidal or hobnail epithelium; and (5) blastemal cells are present, in any amount, with or without other embryonal stromal or epithelial cell types.^7^ Criteria (3) and (5) are useful to distinguish CPDN from both CN and Wilms tumor since CN meets criteria (1) to (4) and Wilms tumor typically contains solid nodules. Histopathological findings in our case met the criteria for the diagnosis of CPDN.

However, immunohistological criteria for the diagnosis of CPDN have not been defined yet. In the adult CPDN cases, only one case was positive for WT1 protein, which is positive in most cases of Wilms tumor.^3^, ^4^ Therefore, WT1 protein staining is unable to differentiate CPDN from Wilms tumor. However, immunohistological examination did help to identify blastemal cells, which are essential to definitively diagnose CPDN.^4^, ^8^ Further studies are needed to establish the immunohistological criteria.

The standard treatment for CPDN in children is surgical resection.^1^, ^3^, ^6^ Although CPDN is a cystic variant of Wilms tumor, which is malignant, CPDN has limited potential for invasion and metastasis, resulting in an excellent prognosis.^9^ However, adjuvant chemotherapy can be taken into consideration for obtaining a better survival outcome in cases where the tumor extends beyond the kidney or the margin of resection.^10^ In the present case, radical nephrectomy was performed since partial nephrectomy would raise the risk of complications due to the tumor location. Based on the pathological findings, adjuvant chemotherapy was not performed.

No recurrence occurred after surgery in at least three out of the five adult CPDN cases (Table 1), suggesting surgical resection can lead to a cure. Furthermore, no recurrence occurred during 13 years of follow‐up with our patient, which indicates surgical resection can be used as an established treatment option in adult cases as well as in children. However, recurrence with tumor spillage during surgery has been reported in children cases.^1^ Therefore, complete resection should be achieved as well as in other malignant kidney tumors, regardless whether a total or partial nephrectomy is performed.

## Conclusion

We encountered the oldest adult patient with CPDN. CPDN can develop in adults, regardless of age. Since preoperative discrimination of CPDN from other multicystic tumors is difficult, histopathological examination is needed for a definitive diagnosis. Though immunohistological criteria for the diagnosis of CPDN has not been developed yet, immunohistological examination could contribute to confirm a diagnosis of CPDN. Surgical resection can treat CPDN in adults, as demonstrated in the present case where no recurrence was observed for 13 years after the operation. Complete resection should be accomplished to prevent recurrence.

## Conflict of interest

The authors declare no conflict of interest.

## Approval of the research protocol by an institutional reviewer board

Not applicable.

## Informed consent

Written informed consent was obtained.

## Registry and the registration no. of the study/trial

Not applicable.
